# A 3D Model of the Membrane Protein Complex Formed by the White Spot Syndrome Virus Structural Proteins

**DOI:** 10.1371/journal.pone.0010718

**Published:** 2010-05-19

**Authors:** Yun-Shiang Chang, Wang-Jing Liu, Cheng-Chung Lee, Tsung-Lu Chou, Yuan-Ting Lee, Tz-Shian Wu, Jiun-Yan Huang, Wei-Tung Huang, Tai-Lin Lee, Guang-Hsiung Kou, Andrew H.-J. Wang, Chu-Fang Lo

**Affiliations:** 1 Department of Molecular Biotechnology, Da-Yeh University, Changhua, Taiwan; 2 Institute of Zoology, National Taiwan University, Taipei, Taiwan; 3 Institute of Biological Chemistry, Academia Sinica, Taipei, Taiwan; University of Minnesota, United States of America

## Abstract

**Background:**

Outbreaks of white spot disease have had a large negative economic impact on cultured shrimp worldwide. However, the pathogenesis of the causative virus, WSSV (whit spot syndrome virus), is not yet well understood. WSSV is a large enveloped virus. The WSSV virion has three structural layers surrounding its core DNA: an outer envelope, a tegument and a nucleocapsid. In this study, we investigated the protein-protein interactions of the major WSSV structural proteins, including several envelope and tegument proteins that are known to be involved in the infection process.

**Principal Findings:**

In the present report, we used coimmunoprecipitation and yeast two-hybrid assays to elucidate and/or confirm all the interactions that occur among the WSSV structural (envelope and tegument) proteins VP51A, VP19, VP24, VP26 and VP28. We found that VP51A interacted directly not only with VP26 but also with VP19 and VP24. VP51A, VP19 and VP24 were also shown to have an affinity for self-interaction. Chemical cross-linking assays showed that these three self-interacting proteins could occur as dimers.

**Conclusions:**

From our present results in conjunction with other previously established interactions we construct a 3D model in which VP24 acts as a core protein that directly associates with VP26, VP28, VP38A, VP51A and WSV010 to form a membrane-associated protein complex. VP19 and VP37 are attached to this complex via association with VP51A and VP28, respectively. Through the VP26-VP51C interaction this envelope complex is anchored to the nucleocapsid, which is made of layers of rings formed by VP664. A 3D model of the nucleocapsid and the surrounding outer membrane is presented.

## Introduction

White spot syndrome virus (WSSV; genus *Whispovirus*, family *Nimaviridae*) [1] is a widely occuring virus which attacks cultured shrimp and many other crustaceans and has caused severe mortalities and huge economic losses to the shrimp farming industry globally for more than a decade [2]–[4]. WSSV is a large enveloped virus of approximately 275 by 120 nm in size with an olivaceous-to-bacilliform shape [5]. The WSSV virion consists of four components: a ∼300 kbp double-stranded DNA genome, a nucleocapsid, a tegument, and an outer envelope [6], [7]. The protein components of the WSSV virion have been established by proteomic methods, and at least 58 structural proteins are currently known, over 30 of which are recognized as envelope proteins [6]–[10].

The structural proteins often play vital roles in cell targeting, virus entry, assembly and budding [11]–[15] as well as triggering host antiviral defenses [16]. Some of the WSSV envelope proteins involved in shrimp infection have also been identified [17]–[22], and it has been suggested that they might be used to produce neutralizing antibodies or as targets for vaccine design [17], [19]–[25]. Interactions between structural proteins are common in enveloped viruses, but to date, protein-protein interactions have been reported only in nine WSSV virion proteins, ie VP19, VP24, VP26, VP28, VP37 (also known as VP281), VP38A (also known as VP38), VP51C (also known as VP51), VP51A and WSV010 [10], [22], [26]–[31]. Moreover, most of these studies only investigated the interactions between pairs of proteins, so that even now, little is known about the organization and functional connections that occur in larger, more complex configurations of these components.

Much of the work in the present study centers around the novel WSSV envelope protein VP51A. VP51A corresponds to open reading frame 294 (ORF294) of the WSSV-TW isolate, and it was originally identified in our previous study [26]. Although the precise function of VP51A is not yet clear, the fact that a large portion of its C terminal is exposed outside of the WSSV envelope suggests that it may play an important role in virus infection. In general, protein domains exposed on the surface of viruses play fundamental roles in infection by binding to cell receptors, promoting cell fusion processes, or interacting with elements of the host immune system [11], [15], [16], [32]. VP51A has also been shown to interact with VP26, which in turn interacts with VP28. In the resulting VP51A-VP26-VP28 complex, both VP51A and VP28 are externally exposed [26]. VP28 is a major envelope protein that is implicated in cell attachment during infection [17], [19], [33]. VP26 is a tegument protein [7] (or a matrix-like linker protein between the viral envelope and nucleocapsid [30]) and it has been hypothesized that it may be instrumental in trafficking the WSSV nucleocapsid into the host nucleus via the cytoskeleton [34]. With its protruding C terminus, VP51A may therefore contribute to the functionality of either or both of these two major WSSV structural proteins, as well as potentially being directly involved in host cell recognition or attachment [26]. Two other proteins, VP19 and VP24, are also abundant in the envelope [9], [35]. In this study, interactions between VP51A and these four major envelope/tegument proteins were screened using a matrix approach. By using coimmunoprecipitation, yeast two-hybrid, and chemical cross-linking assays the relationship among these proteins and the oligomerization status of VP19, VP24 and VP51A were defined. From these data, a 3D model was constructed. Lastly, based on additional external data, four other structural proteins, VP37, VP38A, WSV010 and VP51C, were also incorporated into this 3D structural model. We also look at the WSSV nucleocapsid, which consists of a series of 14–15 stacked rings [5], [36], [37]. It is already known that those stacked rings are formed by the major nucleocapsid protein VP664 [38], and here we elaborate a more precise model of VP664's structural role.

## Results

### VP51A interacts with VP19

**Figure 1:**
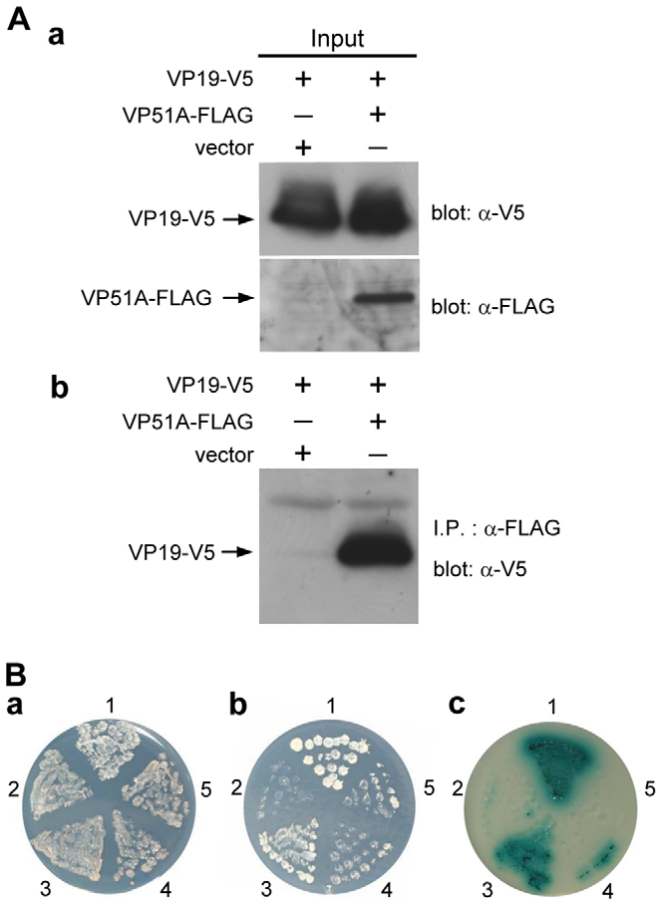
VP51A interacts with VP19. (A) Coimmunoprecipitation of V5-tagged VP19 (VP19-V5) with FLAG-tagged VP51A (VP51A-FLAG) from transfected cells. Sf9 cells were transfected with plasmids expressing VP19-V5, VP51A-FLAG or empty plasmid (vector) as indicated. At 6 h after heat shock, the cell lysates were harvested. (a) After separation by SDS-PAGE, input expression was confirmed by Western blotting using either anti-V5 antibody or anti-FLAG antibody as a probe. Arrows indicate the expressed VP19-V5 and VP51A-FLAG. (b) The cell lysates were immunoprecipitated with anti-FLAG M2 affinity resins and then the immunoprecipitated complexes were subjected to Western blot analysis with an anti-V5 antibody probe. (B) The yeast two-hybrid results confirmed that VP51A specifically interacted with VP19. (a) Yeast growth on medium lacking both Leu and Trp indicates the presence of each respective pair of plasmids. (b) & (c) Yeast growth on low stringency (-Leu/-Trp/-His) and high stringency (-Leu/-Trp/-His/-Ade) medium, respectively. The blue signal in (c) is due to the presence of X-α-Gal. The positive signals represent protein-protein interactions. The numbers around the plate indicate the bait and prey plasmids of the transformed yeast: 1, pGBKT7-53/pGADT7-RecT; 2, pGBKT7-Lam/pGADT7-RecT; 3, pGBK-VP51A/pGAD-VP19; 4, pGBK-VP51A/pGADT7; 5, pGBKT7/pGAD-VP19.

Interactions between VP51A and VP19 were investigated using a coimmunoprecipitation assay in which FLAG-tagged VP51A (VP51A-FLAG) and V5-tagged VP19 (VP19-V5) were coexpressed in Sf9 insect cells. As shown in Figure 1A panel a, both VP51A-FLAG and VP19-V5 were successfully expressed in the Sf9 cells. A pilot experiment confirmed that the VP51A-FLAG proteins could be efficiently precipitated by the anti-FLAG antibody, and binding specificity of VP51A-FLAG with anti-FLAG M2 affinity gel was reconfirmed by subjecting VP51A-FLAG protein to reaction with anti-HA antibody conjugated beads (data not shown). In the coimmunoprecipitation assays, complexes consisting of VP19-V5 plus VP51A-FLAG were coimmunoprecipitated by anti-FLAG M2 affinity gel and detected by Western blotting using anti-V5 antibody (Figure 1A, panel b). A reverse experiment using FLAG-tagged VP19 (VP19-FLAG) and V5-tagged VP51A (VP51A-V5) demonstrated successful expression of these two inputs and produced the expected coimmunoprecipitation results (data not shown). From these results, we conclude that the interaction between VP51A and VP19 is specific and independent of the tags. The interaction between VP51A and VP19 was also investigated using a yeast two-hybrid assay. All of the experimental constructs were able to express successfully in yeast cells (data not shown), and panel a in Figure 1B shows that all of the cotransformants were also able to grow on the SD/-Leu/-Trp plate. In contrast, in the three or four dropout plates, no growth was observed for the pairs of transforming constructs pGBK-VP51A/pGADT7 and pGBKT7/pGAD-VP19, or for the negative control (Figure 1B, panels b and c). Growth on the low stringency (SD/-Leu/-Trp/-His) and high stringency (SD/-Leu/-Trp/-His/-Ade/X-α-Gal) plates was only observed when the yeast was transformed with pGBK-VP51A/pGAD-VP19 or with the positive control (Figure 1B, panels b and c). These results confirmed that VP51A interacts with VP19.

### VP51A interacts with VP24; VP24 interacts with VP26

**Figure 2:**
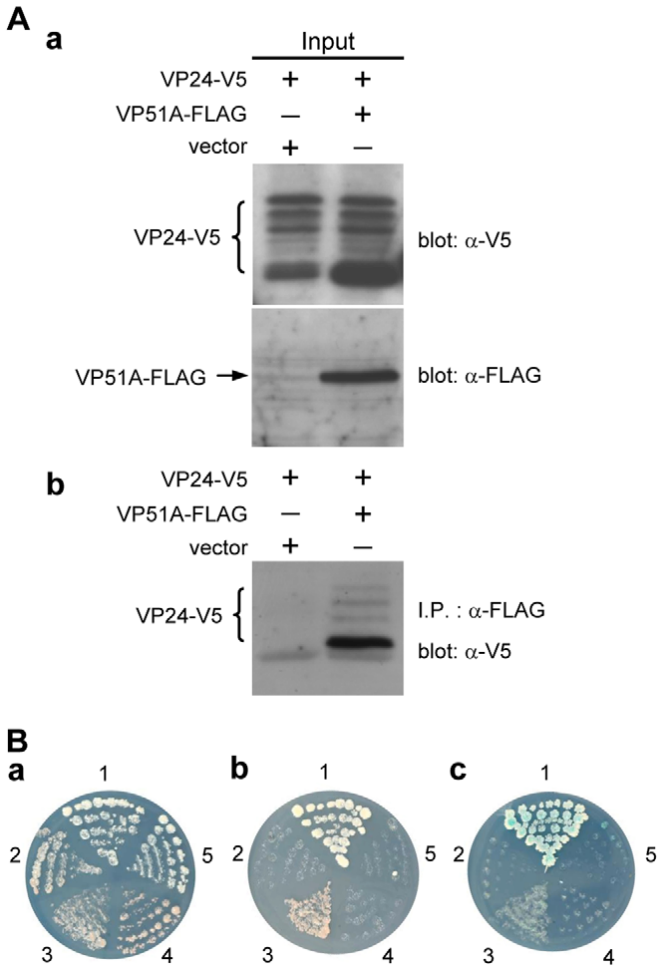
VP51A interacts with VP24. (A) (B) Results are analogous to those described in the legend for Figure 1. VP24-V5 was detected in several bands as indicated by the braces. The numbers around the plates in (B) indicate the bait and prey plasmids of the transformed yeast: 1, pGBKT7-53/pGADT7-RecT; 2, pGBKT7-Lam/pGADT7-RecT; 3, pGBK-VP51A/pGAD-VP24; 4, pGBK-VP51A/pGADT7; 5, pGBKT7/pGAD-VP24.

**Figure 3:**
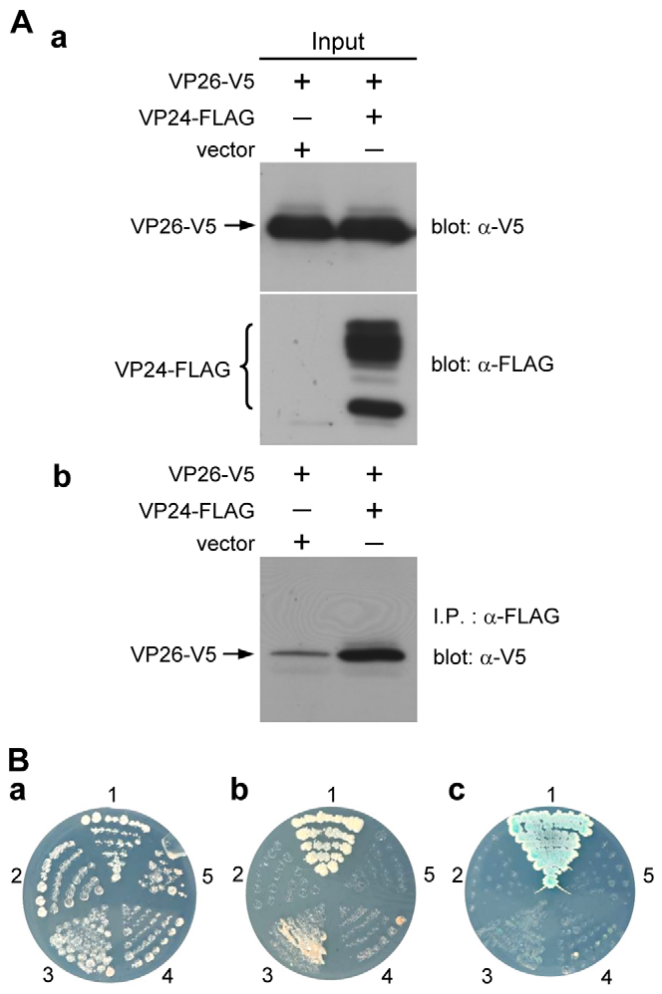
VP26 interacts with VP24. (A) (B) Results are analogous to those described in the legend for Figure 1. VP24-FLAG was detected in several bands as indicated by the brace. The numbers around the plates in (B) indicate the bait and prey plasmids of the transformed yeast: 1, pGBKT7-53/pGADT7-RecT; 2, pGBKT7-Lam/pGADT7-RecT; 3, pGBK-VP24/pGAD-VP26; 4, pGBK-VP24/pGADT7; 5, pGBKT7/pGAD-VP26.

Similar batteries of assays successfully demonstrated interactions between V5- or FLAG-tagged VP51A and tagged VP24 (Figure 2), and between tagged VP24 and tagged VP26 (Figure 3). (VP24 was detected in multiple bands. This is probably due to post-translational modifications; please see the Discussion.) We note however that the blue X-α-Gal signal is much fainter in panel c of Figures 2B and 3B.

A similar battery of coimmunoprecipitation assays failed to demonstrate any interaction between tagged VP24 and tagged VP19 (data not shown).

### Confirmation that VP24 interacts with VP28

**Figure 4:**
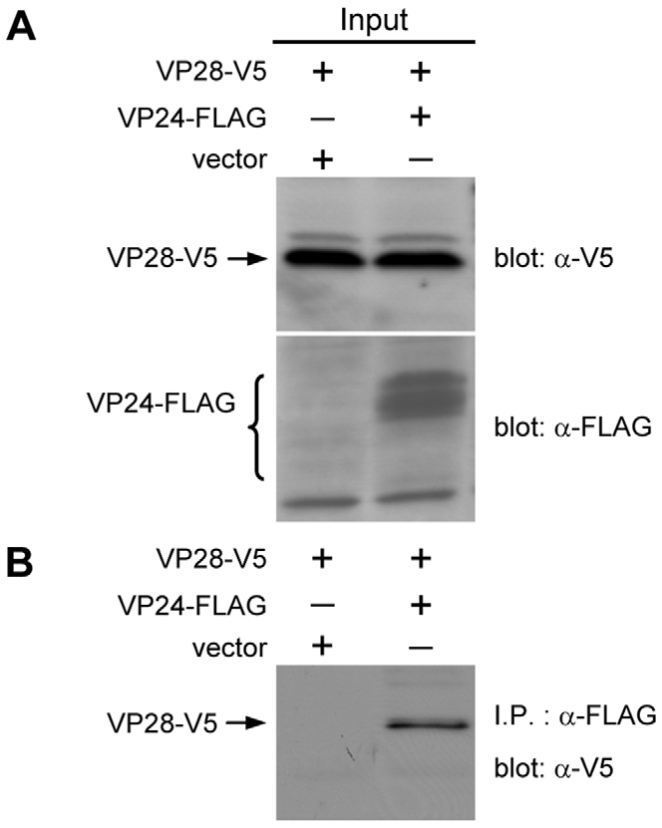
Confirmation of the interaction between VP24 with VP28. (A) (B) Results are analogous to those described in the legend for Figure 1A (a) and (b), respectively. VP24-FLAG was detected in several bands as indicated by the brace.

Interaction between VP24 and VP28 was identified previously by Far-Western analysis [22]. The interaction between these two proteins was confirmed here by coimmunoprecipitation assay. In this assay, the inputs, VP24-FLAG and V5-tagged VP28 (VP28-V5), were successfully expressed in the Sf9 insect cells (Figure 4A), and the VP28-V5 was coimmunoprecipitated with VP24-FLAG (Figure 4B; the absence of one of VP24's multiple bands is probably due to low expression levels, as indicated by the faintness of the other bands). A reverse experiment using FLAG-tagged VP28 (VP28-FLAG) and VP24-V5 produced the same results (data not shown).

**Figure 5:**
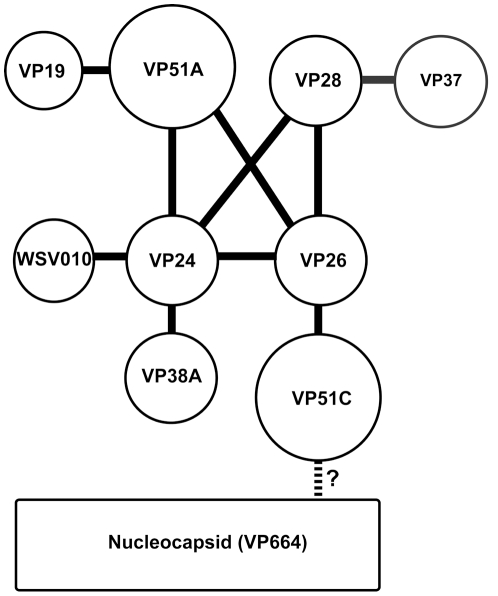
A matrix diagram showing the relationships between each of the studied proteins. VP24 associates directly with VP26, VP28, VP38A, VP51A and WSV010, and it acts as the core of a protein complex. VP19 associates with the complex via VP51A only. VP37 associates with the complex via VP28. VP26 has a direct interaction with the nucleocapsid protein VP51C. Although the interactions among VP664, VP51C and other structural proteins are not yet fully understood, it is likely that the entire envelope protein complex is able to anchor on the nuclocapsid via an interaction between VP51C and VP664.

Based on the protein-protein interactions listed above and reported previously, the relationships among VP19, VP26, VP28, VP37, VP38A, VP51A, VP51C and WSV010 are summarized in a matrix diagram (Figure 5).

### VP19, VP24 and VP51A have an affinity for self-interaction

**Figure 6:**
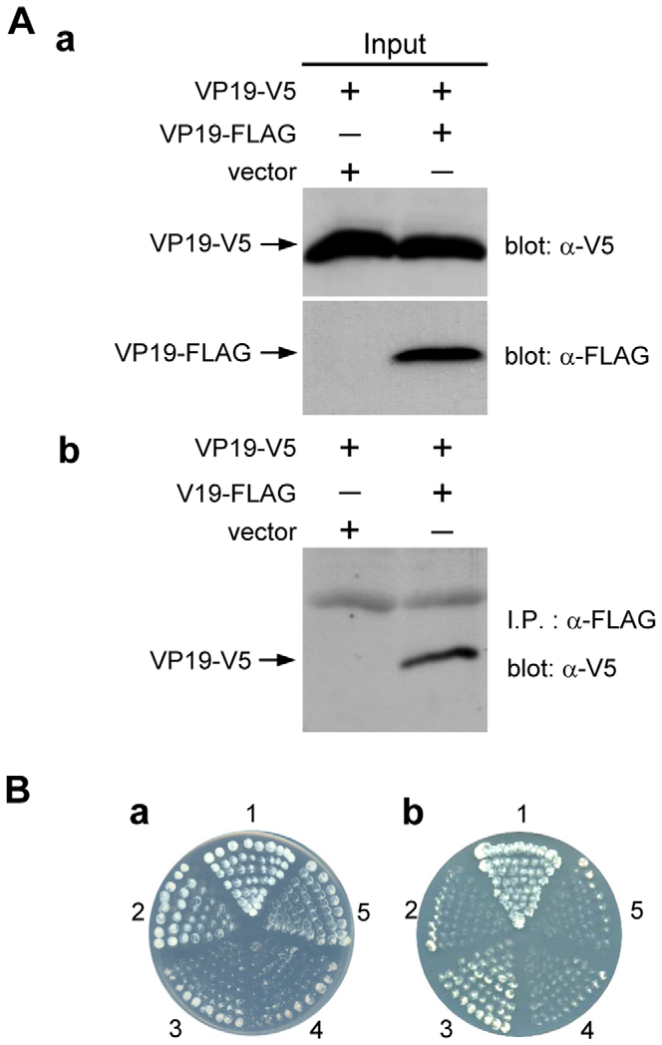
Self-interaction of VP19. (A) Coimmunoprecipitation of V5-tagged VP19 (VP19-V5) with FLAG-tagged VP19 (VP19-FLAG) from transfected cells. Sf9 cells were transfected with plasmids expressing VP19-V5, VP19-FLAG or empty plasmid (vector) as indicated, and at 6 h after heat shock, the cell lysates were harvested. (a) Lysates were separated by SDS-PAGE and input expression was confirmed by Western blotting using either anti-V5 antibody or anti-FLAG antibody as a probe. Arrows indicate the expressed VP19-V5 and VP19A-FLAG. (b) The cell lysates were immunoprecipitated with anti-FLAG M2 affinity resins and then the immunoprecipitated complexes were subjected to Western blot analysis with an anti-V5 antibody probe. (B) The yeast two-hybrid results confirmed that VP19 interacted with itself. (a) Yeast growth on medium lacking both Leu and Trp indicates the presence of each respective pair of plasmids. (b) Yeast growth on low stringency (-Leu/-Trp/-His). The positive signals represent protein-protein interactions. The numbers around the plate indicate the bait and prey plasmids of the transformed yeast: 1, pGBKT7-53/pGADT7-RecT; 2, pGBKT7-Lam/pGADT7-RecT; 4. pGBK-VP19/pGADT75. pGBKT7/pGAD-VP19; 3, pGBK-VP19/pGAD-VP19.

**Figure 7:**
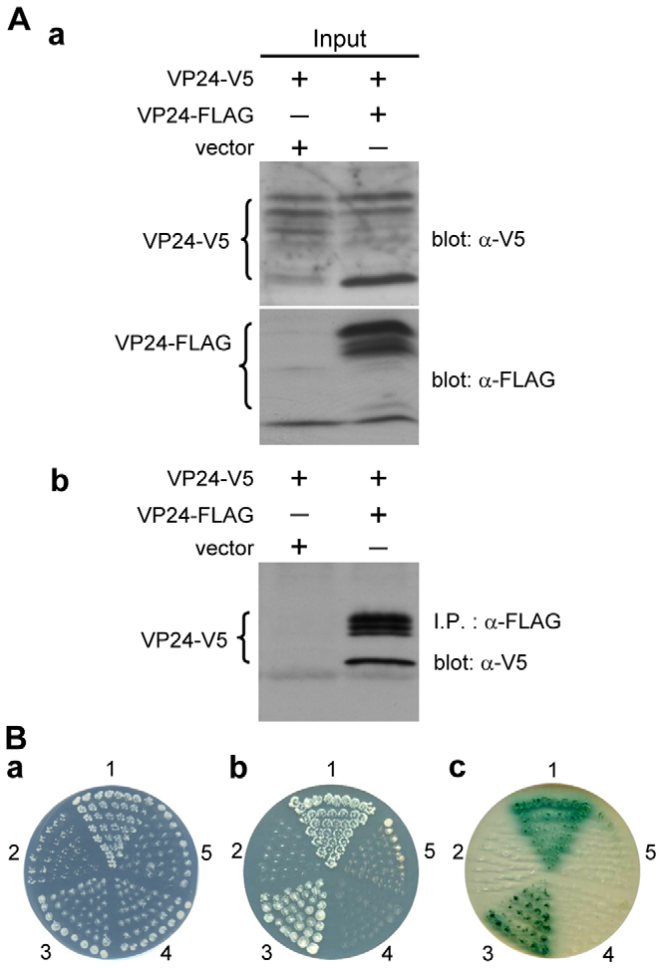
Self-interaction of VP24. (A) (B) Results are analogous to those described in the legend for Figure 6. VP24-V5 and VP24-FLAG were detected in several bands as indicated by the braces. The additional panel c in Figure 7B shows yeast grown under high-stringency conditions. The numbers around the plate indicate the bait and prey plasmids of the transformed yeast: 1, pGBKT7-53/pGADT7-RecT; 2, pGBKT7-Lam/pGADT7-RecT; 3, pGBK-VP24/pGAD-VP24; 4, pGBK-VP24/pGADT7; 5. pGBKT7/pGAD-VP24.

**Figure 8:**
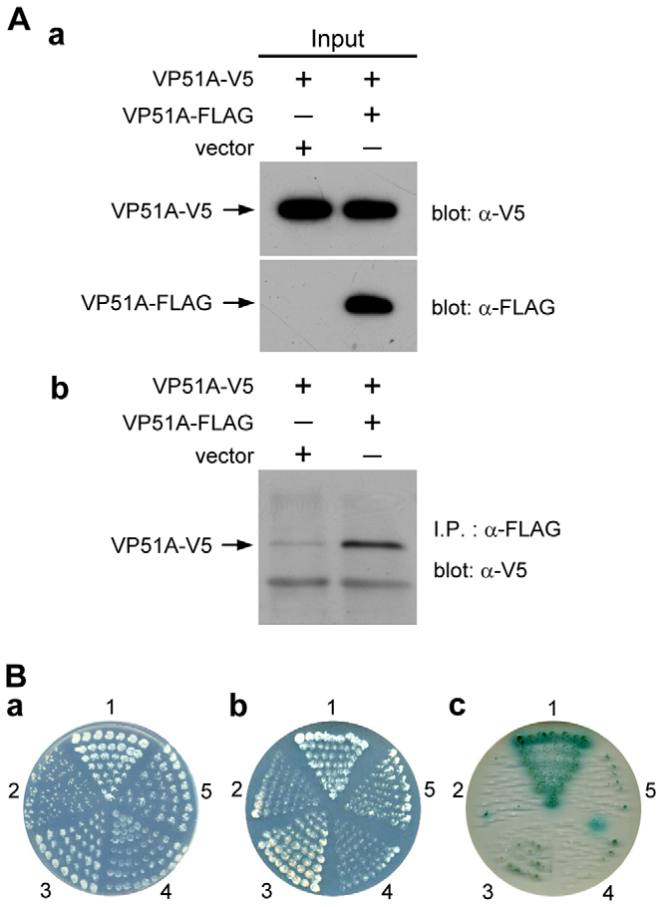
Self-interaction of VP51A. (A) (B) Results are analogous to those described in the legend for Figure 6. The additional panel c in Figure 8B shows yeast grown under high-stringency conditions. The numbers around the plates indicate the bait and prey plasmids of the transformed yeast: 1, pGBKT7-53/pGADT7-RecT; 2, pGBKT7-Lam/pGADT7-RecT; 3, pGBK-VP51A/pGAD-VP51A; 4, pGBK-VP51A/pGADT7; 5, pGBKT7/pGAD-VP51A.

WSSV VP26 and VP28 have already been shown to self-interact to form trimers [39]. Here we demonstrated the self-interaction of VP19, VP24 and VP51A by *in vitro* biochemical binding assays. In the coimmunoprecipitation assays for the self-interaction of VP19, VP24 and VP51A, the inputs, VP19-V5, VP19-FLAG, VP24-V5, VP24-FLAG, VP51A-V5 and VP51A-FLAG were successfully expressed in the Sf9 insect cells and each of the V5-tagged protein was coimmunoprecipitated with appropriate FLAG-tagged protein (VP19-V5 with VP19-FLAG, VP24-V5 with VP24-FLAG and VP51A-V5 with VP51A-FLAG) (Figures 6A, 7A and 8A). Yeast two-hybrid assays also confirmed the self-interactions listed above. After checking that all of the constructs were able to express successfully in yeast cells (data not shown), we found that all of the cotransformants were able to grow on the SD/-Leu/-Trp plates (panel a of Figures 6B, 7B and 8B). There was no reporter gene activation in the control pairs pGBKT7/pGAD-VP19, pGBKT7/pGAD-VP24, pGBKT7/pGAD-VP51A, pGBK-VP19/pGADT7, pGBK-VP24/pGADT7, and pGBK-VP51A/pGADT7 or in the negative controls (Figure 6B, panel b; panels b and c of Figures 7B and 8B). Growth on the low stringency and high stringency plates was only observed when the yeast was transformed with pGBK-VP24/pGAD-VP24, pGBK-VP51A/pGAD-VP51A or with the positive control (panels b and c of Figures 7B and 8B). The pGBK-VP19/pGAD-VP19 transformed yeast grew successfully on the low stringency plate (Figure 6B, panel b) but not on the high stringency medium (data not shown). From these results, we conclude that VP19, VP24 and VP51A undergo specific self-interaction.

### VP19, VP24 and VP51A form dimers

**Figure 9:**
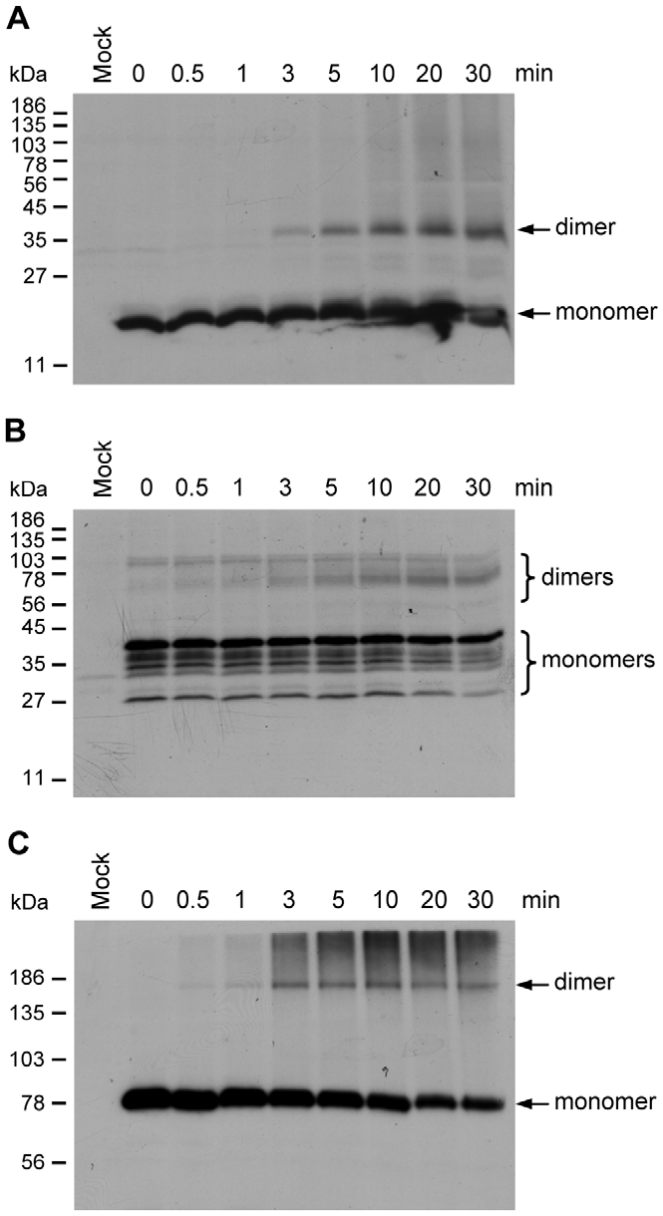
Oligomerization status of VP19, VP24 and VP51A. (A) Kinetic study of glutaraldehyde cross-linking of (A) VP19, (B) VP24 and (C) VP51A expressed in Sf9 cells. Transiently expressed V5-tagged VP19, VP24 and VP51A. were treated with 0.01% glutaraldehyde for the indicated times at room temperature and subjected to Western blot with anti-V5 antibody. Mock: transfection with vector plasmid.

A chemical cross-linking assay was used to investigate the forms of the VP19, VP24 and VP51A oligomerization. For the cross-linking study, Sf9 cells were transfected with expression plasmid that contained the full-length VP19, VP24 or VP51A coding region under control of *Drosophila* heat shock protein 70 promoter. The cellular lysates were cross-linked with glutaraldehyde, and the recombinant proteins were detected by immunoblotting using anti-V5 antibody. With increasing treatment time, there was a steady accumulation of dimeric-sized VP19, VP24 and VP51A (Figures 9A, B and C). As the amounts of dimeric polypeptide increased, there was a corresponding decrease in the amount of monomeric VP19, VP24 and VP51A.

### Identification of the interaction domains of VP19, VP24, VP26, VP28 and VP51A

**Figure 10:**
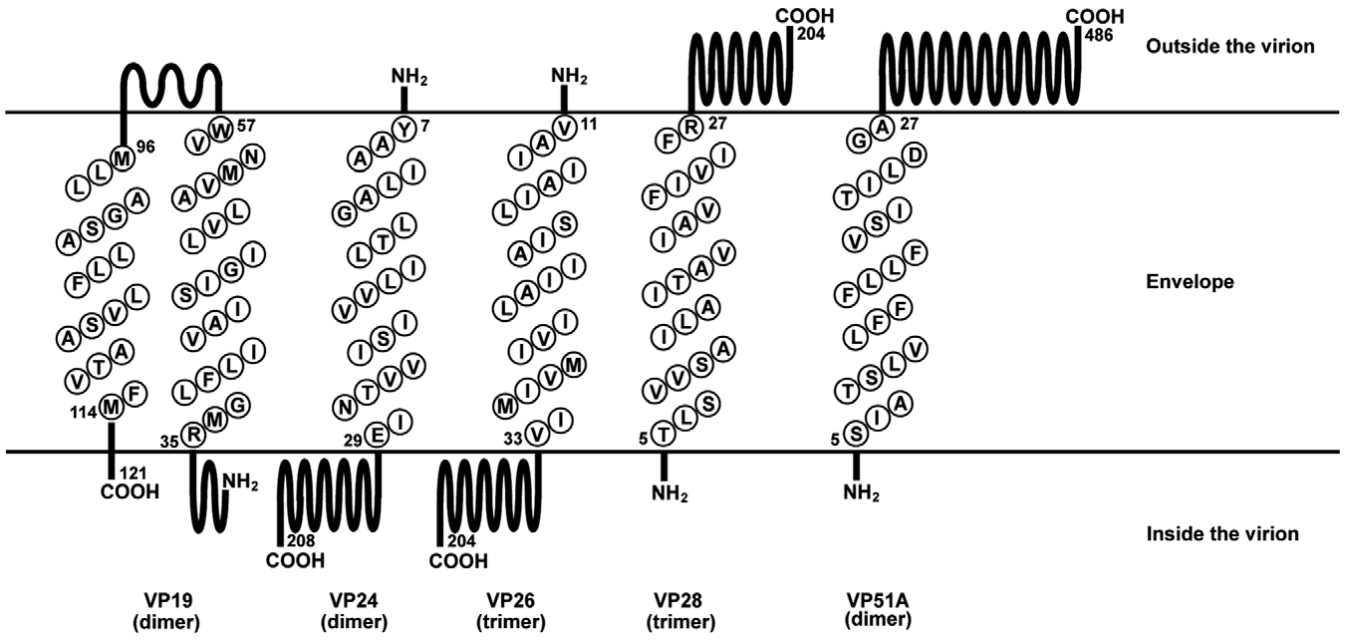
SOSUI predictions of the membrane topology of VP19, VP24, VP26, VP28 and VP51A. The single letter amino acid sequences embedded in the envelope region correspond to the transmembrane helices of each protein. The C-terminus orientation of VP24 was left undecided by the SOSUI program, and its location was therefore inferred from other data [7], [27], [40].

Based on Table 1, which summarizes the pairwise interactions that are known to occur between these five proteins, batteries of coimmunoprecipitation assays were performed between the corresponding FLAG tagged full length structural proteins and V5 tagged partial proteins. Table 2 shows the results of these assays. From these results, we infer that: the N-terminal region of VP19 interacts with the N-terminal region of VP51A; both the N- and C-terminals of VP24 interacted with the VP26 and VP28 N-terminals; VP26 interacted with the N-terminal and middle fragment of VP51A. Some results were inconsistent. For example, the full length VP51A interacted with both the N- and C-terminal of VP24, even though none of the VP51A partial fragments interacted with full length VP24. We also found that both the N- and C-terminals of VP26 interacted with the N- and C-terminals of VP28, although their predicted *in vivo* configuration (see Figure 10 below) would make it almost impossible for the C-terminal of VP26 to interact with the C-terminal of VP28. The self-interaction results showed that: the full length VP24 and VP26 proteins only interacted with their N-terminal regions; the full length VP19 and VP28 proteins interacted with both their N- and C-terminals; the full length VP51A protein interacted with its N-, C-, and mid-region partial fragments.

**Table 1:** Known interactions between WSSV structural proteins.

		VP19	VP24	VP26	VP28	VP51A
envelope (or tegument)	VP19	+				
	VP24	−/+^31^	+^31^			
	VP26	−	+^31^	+^39^		
	VP28	−/+^31^	+^22^	+^9^	+^39^	
	VP51A	+	+	+^26^	−^26^	+
	VP37				+^28^	
	VP38A		+^29^	−^29^		
	WSV010		+^27^			
nucleocapsid	VP51C			+^30^		

Known interactions are indicated with a plus sign (+). A minus sign (−) indicates that the proteins do not interact.

Superscript numbers indicate the reference for previously published data.

−/+^31^: Zhou et al., (2009) reported that VP19 interacted with VP24 and VP28, but these interactions were not observed in the present study.

**Table 2:** Identification of interaction domains.

Construction (full length)	Construction (partial fragment)	Interaction*
pDHsp/VP19-FLAG-His	pDHsp/EGFP-VP19_1–66_-V5-His	+
	pDHsp/EGFP-VP19_62–121_-V5-His	+
	pDHsp/EGFP-VP51A_1–165_-V5-His	+
	pDHsp/EGFP-VP51A_168–333_-V5-His	−
	pDHsp/EGFP-VP51A_326–488_-V5-His	−
pDHsp/VP24-FLAG-His	pDHsp/EGFP-VP24_1–105_-V5-His	+
	pDHsp/EGFP-VP24_99–208_-V5-His	−
	pDHsp/EGFP-VP26_1–97_-V5-His	+
	pDHsp/EGFP-VP26_85–204_-V5-His	−
	pDHsp/EGFP-VP28_1–133_-V5-His	+
	pDHsp/EGFP-VP28_110–204_-V5-His	−
	pDHsp/EGFP-VP51A_1–165_-V5-His	−
	pDHsp/EGFP-VP51A_168–333_-V5-His	−
	pDHsp/EGFP-VP51A_326–488_-V5-His	−
pDHsp/VP26-FLAG-His	pDHsp/EGFP-VP24_1–105_-V5-His	+
	pDHsp/EGFP-VP24_99–208_-V5-His	+
	pDHsp/EGFP-VP26_1–97_-V5-His	+
	pDHsp/EGFP-VP26_85–204_-V5-His	−
	pDHsp/EGFP-VP28_1–133_-V5-His	+
	pDHsp/EGFP-VP28_110–204_-V5-His	+
	pDHsp/EGFP-VP51A_1–165_-V5-His	+
	pDHsp/EGFP-VP51A_168–333_-V5-His	+
	pDHsp/EGFP-VP51A_326–488_-V5-His	−
pDHsp/VP28-FLAG-His	pDHsp/EGFP-VP24_1–105_-V5-His	+
	pDHsp/EGFP-VP24_99–208_-V5-His	+
	pDHsp/EGFP-VP26_1–97_-V5-His	+
	pDHsp/EGFP-VP26_85–204_-V5-His	+
	pDHsp/EGFP-VP28_1–133_-V5-His	+
	pDHsp/EGFP-VP28_110–204_-V5-His	+
pDHsp/VP51A-FLAG-His	pDHsp/EGFP-VP19_1–66_-V5-His	+
	pDHsp/EGFP-VP19_62–121_-V5-His	−
	pDHsp/EGFP-VP24_1–105_-V5-His	+
	pDHsp/EGFP-VP24_99–208_-V5-His	+
	pDHsp/EGFP-VP26_1–97_-V5-His	+
	pDHsp/EGFP-VP26_85–204_-V5-His	+
	pDHsp/EGFP-VP51A_1–165_-V5-His	+
	pDHsp/EGFP-VP51A_168–333_-V5-His	+
	pDHsp/EGFP-VP51A_326–488_-V5-His	+

A plus sign (+) indicates that the expressed proteins interacted; a minus sign (−) indicates that they did not.

### Modeling the structural protein complex composed of VP19, VP24, VP26, VP28, VP37, VP38A, VP51A, VP51C and WSV010

**Figure 11:**
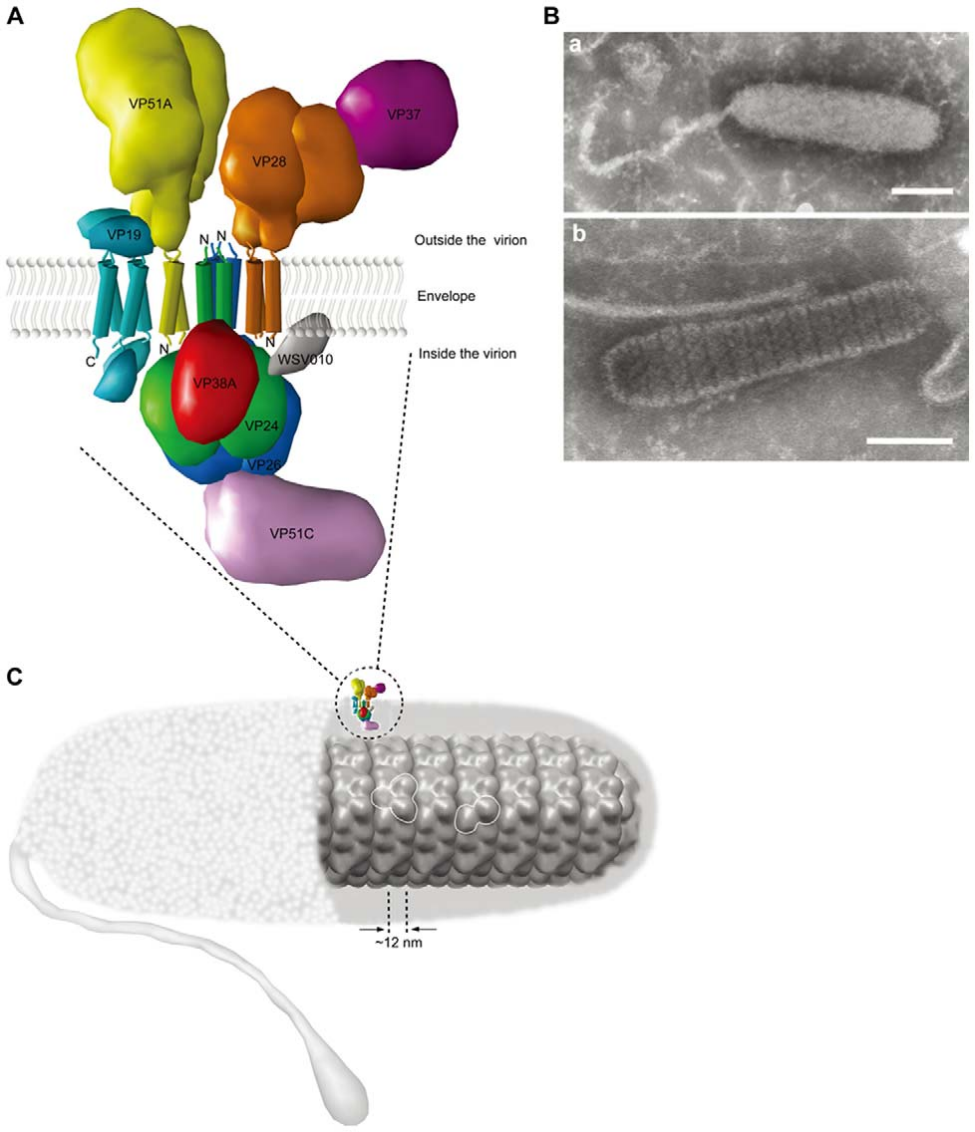
3D models of the membrane protein complex and the nucleocapsid. (A) Graphical representation of the putative relative localizations and interactions of the WSSV virion protein complex composed of VP19, VP24, VP26, VP28, VP37, VP38A, VP51A, VP51C and WSV010. Each protein is rendered using a unique color. The transmembrane helices are shown as cylinders. The oligomerization of VP19, VP24, VP51A (dimers), VP26 and VP28 (trimers) is also shown. (B) Electron micrographs of a negatively stained (a) intact WSSV virion and (b) WSSV nucleocapsid. Bars in both panels are 100 nm. (C) Refined model of the WSSV virion nucleocapsid. Each globular shape is ∼12 nm in diameter and corresponds to a single VP664 molecule. Dimers or trimers of VP664 (outlined in white) are arranged into the stacked rings that can be seen under the electron microscope. Note that the insert of the protein cluster in only intended to show its location and orientation; it is not drawn to scale.

Membrane topology predictions for VP19, VP24, VP26, VP28 and VP51A are shown in Figure 10. All three prediction programs displayed only very slight variations for each protein, through different prediction methods gave different start and end points for the predicted transmembrane segments. According to the prediction results of the SOSUI program, VP19 contains two consensus helices at amino acids 35–57 and 96–114, and these are connected by an outer membrane loop. VP24, VP26, VP28 and VP51A all have very similar prediction results. Each of these proteins has a transmembrane helix of 23 amino acids at its N-terminus. The C-terminals of VP28 and VP51A were predicted to be exposed outside of the virion envelope, and for VP51A, this topological prediction is consistent with previous experimental results [26]. The predicted location of the C-terminal of VP26 was inside the virion. The location of the C-terminal of VP24 was undecided; it was predicted to be either inside or outside of the envelope with equal probability (data not shown). However, other evidence ([7], [27], [40]; see Discussion) suggests that the C-terminus of VP24 projects inward, and thus it is shown inside the virion in Figure 10. The above topological predictions, the known protein-protein interactions (Table 1; interactions for the proteins VP37, VP38A, WSV010 and VP51C are also included), and the interaction domains (Table 2) plus evidence that VP26 and VP28 both exist in trimeric form [39] were then used to construct a 3D model of the viral protein complex (Figure 11A).

### Refining the nucleocapsid model

Negative-stain electron microscopy shows that in the absence of the envelope, the WSSV nucleocapsid consists of a series of 14–15 stacked rings [5], [36], [37] (Figure 11B panel b). However, the nucleocapsid's ultrastructure has not yet been clearly resolved. The stacked rings are formed by the nucleocapsid protein VP664 [38], but until now it has not been clear exactly how the protein molecules were related to the regular arrangement of globular shapes that made up each ring. Based on the observed mass (644 kDa) of VP664, the size-mass relationship found by Erickson [41] predicts a protein diameter of about 11.5 nm with a minimal radius (*R*_min_) of 5.76 nm. Since the observed globular shapes are about 12 nm in diameter (Figure 11B panel b), this suggests that each globular shape corresponds to a single VP664 molecule (Figure 11C). Further, since the arrangement of the globules exhibits a 2- or 3-fold symmetry, it is likely that each ring may in fact be composed of a number of repeats of the dimeric or trimeric form (18 dimers or 12 trimers) of VP664 (white outlines in Figure 11C).

## Discussion

Based on our protein-protein results (Figures 2, 3 and 4) as well as previous results for WSV010 [27] and VP38A [29], the model that we have constructed has VP24 acting as the core of a protein complex which is formed by direct association of VP26, VP28, VP38A, VP51A and WSV010 (Figure 5). VP19 and VP37 are also part of this protein complex, but their reported protein-protein interactions are not entirely consistent. Our coimmunoprecipitation analysis revealed that VP19 did not react with VP24, VP26 or VP28 (data not shown), but a recent report [31] indicated that VP19 associates with both VP24 and VP28. Meanwhile, in addition to forming dimers (Figure 9), VP19 was also shown to interact with VP51A (Figure 1). Liu et al. [28] reported that VP37 was able to bind to both VP26 and VP28 but their additional assays only confirmed an interaction between VP37 and VP28 and not between VP37 and VP26. Our preferred model at this point is therefore that VP19 and VP37 only associate with the complex via VP51A and VP28, respectively (Figure 5); however, further research will be required to confirm this tentative conclusion. We also note that VP26 has a direct interaction with the nucleocapsid protein VP51C [30]. Although the interactions among VP664, VP51C and other structural proteins are not yet completely known, VP664 and VP51C may associate by direct interaction or else by means of some other proteins/factors. If so, then this would mean that the entire envelope protein complex would be able to anchor on the nuclocapsid via the VP26-VP51C interaction (Figure 5). It is also possible that the tegument proteins VP24 and VP26 might form additional direct links with VP664. Although there is no direct evidence for this at the moment, this possibility seems especially likely for VP24, which shows an affinity for many structural proteins. Lastly, we note that VP26 and VP28 are both major WSSV structural proteins, and together they account for over 60% of the virion's envelope [39]. All of this suggests that the formation of this protein complex and its interactions with other structural proteins are likely to be important for the maintenance of virion structure as well as for the process of virus morphogenesis.

The N-terminal regions of VP19, VP24, VP26, VP28 and VP51A were predicted to be embedded in the viral envelope layer (Figure 10). The interaction domain identification results (Table 2) further suggest that in most cases, these N-terminal regions are also likely to mediate the protein-protein interactions. This is especially true for the interaction between VP19 and VP51A: these two proteins are only associated with each other through their N-termini. For all the proteins, the N-terminus also appears to be important for self-interaction, because all the N-terminal fragments successfully interacted with their own full length proteins (Table 2). Some self-interactions also appear to be mediated by additional sites: all the partial fragments of VP19, VP28 and VP51A were shown to interact with their own full length proteins, which implies that the monomers of these proteins might interact via several sites to form the oligomers. Lastly, we note that the results shown in Table 2 were not sufficient to clearly elucidate the interaction regions of some proteins. Thus for interactions such as VP26/VP28 and VP24/VP51A a more detailed molecular dissection analysis will be needed.

The SOSUI program assigned equal probabilities to whether the C-terminal topology of VP24 would be located on the inside or outside of the envelope. Additional external evidence was therefore used to resolve this question. Immunoelectron microscopy of WSV010, a low-abundance WSSV envelope protein that is associated with VP24, detected positive signals when the envelope was partially disrupted by pretreating the virion with 0.1% Tween 20 [27]. This result suggested that WSV010 is located on the inside surface of the viral envelope. Further evidence comes from an earlier study by Tsai et al. [7], which concluded that VP24 is a tegument protein. In addition, we note that VP24 was also originally thought to be a nucleocapsid protein [40]. We therefore concluded that the C-terminal of VP24 probably protrudes toward the inside of the virion (Figure 10).

Envelope proteins without a transmembrane domain can anchor on the membrane indirectly by interacting with other proteins that do contain a transmembrane domain [42]. As noted above, WSV010 interacts with VP24 [27], and since WSV010 has no predicted transmembrane domain, its association with the envelope is probably mediated by VP24. VP24 is also reported to associate with another low abundance envelope protein, VP38A [29]. VP38A has no predicted transmembrane domain, and it does not associate with VP26 [29], but its interaction with VP24 suggests that it will probably be localized on the inner surface of the virion envelope. VP37 is reported to be an envelope protein that interacts directly with VP28 and which does not have a transmembrane domain [28], [43]. These inferences were used to incorporate WSV010, VP38A and VP37 into the 3D model of the WSSV virion protein complex that is shown in Figure 11A.

Some WSSV structural proteins evidently exist in the virion in oligomeric form. For example, VP26 and VP28 were reported to occur as trimers in the viral envelope [39]. Here we performed *in vitro* biochemical binding assays that suggested that VP19, VP24 and VP51A can also directly self-interact to form dimers (Figures 6, 7, 8 and 9). In the case of VP19, however, the yeast two-hybrid assay results showed that the pGBK-VP19/pGAD-VP19 transformed yeast could grow on the low stringency medium (Figure 6B, panel b) but not on the high stringency medium (data not shown). This implies there is only a weak propensity for the VP19 monomers to form dimers. We also note that the glutaraldehyde cross-linking assay of VP24 revealed several high molecular mass proteins that were larger than expected (Figure 9B). Similar phenomena were also observed in the coimmunoprecipitation results that related to VP24 (Figures 2A, 3A, 4A and 7A). It has been reported previously that at least one WSSV structural protein is glycosylated when expressed in Sf9 insect cells [44], and we therefore speculate that these unexpectedly large proteins might also be due to the recombinant VP24 undergoing post-translational modifications in the Sf9 cells. (It should also be pointed out that while it is usual for the structural proteins of animal viruses to be glycosylated, previous reports show that none of the WSSV structural proteins are glycosylated [35], [44]).

In our model of this WSSV envelope protein complex, three of the proteins with transmembrane domains, VP19, VP28 and VP51A, have large portions exposed on the outer surface of the virion (Figures 10 and 11A). Protein domains on the external surfaces of a virus often play important roles in infection by binding to the cell receptors or promoting the cell fusion process [11], [16]. VP28 has already been implicated in cell attachment during infection [17], [19], [33], and both VP19 and VP51A might also have a similar function. The fourth external protein in this complex, VP37, contains an RGD motif [43]. RGD is a sequence that is associated with cell attachment, and VP37 has been shown to attach to shrimp cell membranes [18], [45]. Among the proteins on the inside of the envelope, VP26 may be instrumental in trafficking the WSSV nucleocapsid into the host nucleus via the cytoskeleton [34]. VP24 has been reported to interact with VP28 and to be involved in virus infection [22]. Large DNA viruses usually have more than one virus attachment protein [46], [47]. During the virus infection process there may be many related proteins that take part. Herpes simplex virus (HSV), for example, initially attaches to the cells by binding the virus proteins gC and gB to the cell receptor heparin sulfate proteoglycans; membrane fusion of HSV is then induced by gD after interacting with additional receptors [48]. There are already reports that WSSV attaches to more than one shrimp cell receptor [32], [49]. All of this suggests that the envelope complex identified in this study may act as an “infectome” which plays a role in cell recognition, as well as in attaching and also guiding the virus into the cell.

## Materials and Methods

### Virus

The WSSV-TW strain was isolated from a batch of WSSV-infected *Penaeus monodon* collected in Taiwan in 1994 [5], [50], and it was used as the template for amplification of the *vp19*, *vp24*, *vp26*, *vp28* and *vp51A* coding regions in all of the following experiments.

### Coimmunoprecipitation

Full-length WSSV VP19, VP24, VP26, VP28 and VP51A genes were inserted into V5- or FLAG-tagged vectors containing the heat inducible *Drosophila* heat shock protein 70 gene promoter (pDHsp/V5-His and pDHsp/FLAG-His [51]) by PCR cloning using WSSV genomic DNA as the template. The primers used for PCR are listed in Table S1. For DNA transfection, Sf9 insect cells were seeded onto a 6-well plate (8×10^5^ cells/well) and grown overnight at 27°C in Sf-900 II serum-free medium (Invitrogen). Using Cellfectin reagent (Invitrogen), each of the V5-tagged plasmids containing the appropriate genes (ie pDHsp/VP19-V5-His, pDHsp/VP24-V5-His, pDHsp/VP26-V5-His, pDHsp/VP28-V5-His and pDHsp/VP51A-V5-His) and the empty vector (pDHsp/V5-His) were cotransfected with one of the FLAG-tagged plasmids (ie pDHsp/VP19-FLAG-His, pDHsp/VP24-FLAG-His, pDHsp/VP26-FLAG-His, pDHsp/VP28-FLAG-His and pDHsp/VP51A-FLAG-His) or with the empty vector (pDHsp/FLAG-His) into the Sf9 cells (2 µg for each plasmid). At 16–18 h after cotransfection, the cells were heat shocked (42°C water bath for 30 min) and then returned to 27°C. At 6 h after heat shock, the cells were washed with 1× phosphate-buffered saline (PBS) and lysed in 100 µl of NP-40 lysis buffer (50 mM Tris-HCl, pH 8.0, 150 mM NaCl, 1% NP-40) supplemented with a protease inhibitor cocktail tablet (Roche). The lysis procedure was carried out on ice for 10 min with occasional shaking. The lysate was centrifuged at 12,000×*g* for 5 min, and an aliquot of the supernatant (10 µl) was reserved to confirm the expression of the transfected genes. The remaining supernatant (90 µl) was then incubated with 15 µl of anti-FLAG M2 affinity gel (Sigma) at 4°C overnight with rotation. The gel was then washed five times in 150 µl of NP-40 lysis buffer. Aliquots of the total cell lysates and immunoprecipitated complexes were separated by sodium dodecyl sulfate-polyacrylamide gel electrophoresis (SDS-PAGE) and transferred to polyvinylidene difluoride (PVDF) membranes (MSI). The membranes were incubated in blocking buffer (5% skim milk in Tris-buffered saline [TBS] [50 mM Tris, 500 mM NaCl, pH 7.5]) at 4°C overnight and then incubated with blocking buffer containing primary antibodies for 1 h at room temperature. Next, the membrane was washed three times with TBS-T (0.5% Tween 20 in TBS), and incubated with a horseradish peroxidase (HRP)-conjugated secondary antibody. After three more washes, the proteins were visualized using a chemiluminescence reagent (Perkin-Elmer, Inc.). V5-tagged fusion proteins were detected with rabbit anti-V5 antibody (Sigma) and goat anti-rabbit immunoglobulin G (IgG)-HRP conjugate (Sigma). FLAG-tagged fusion proteins were detected with rabbit anti-FLAG polyclonal antibody (Sigma) and goat anti- rabbit IgG-HRP conjugate.

### Yeast two-hybrid assay

Protein-protein interaction assays were performed using a commercial yeast two-hybrid system (Matchmaker 3, Clontech) according to the manufacturer's protocol. The prey plasmids pGAD-VP19, pGAD-VP24, pGAD-VP26 and pGAD-VP51A were constructed by respectively cloning the PCR-amplified, full-length VP19, VP24, VP26, and VP51A genes into the pGADT7 in frame with the GAL4 activation domain. The bait plasmids pGBK-VP19, pGBK-VP24 and pGBK-VP51A were constructed by using enzyme digestion to clone the full-length of the indicated genes from the prey plasmids into the pGBKT7 in frame with the GAL4 DNA binding domain. The PCR primer sequences are listed in Table S1. For the protein-protein interaction assay, *Saccharomyces cerevisiae* strain AH109 cells were cotransformed with bait and prey plasmids using the lithium acetate method and plated on selective agar. The proteins were tested for autoactivation by cotransforming their respective plasmids with an empty prey or bait plasmid. The plasmids pGBKT7-53/pGADT7-RecT and pGBKT7-Lam/pGADT7-RecT (supplied with the kit) were also cotransformed into the AH109 cells as positive and negative control, respectively. Following transformation, the AH109 cells were plated onto synthetic dropout (SD) medium lacking leucine (Leu) and tryptophan (Trp) to verify that both of the transformed plasmids were present. To select for yeast that contained interacting proteins, colonies that carried both plasmids were then plated onto SD medium lacking Leu, Trp and histidine (His), and also onto SD medium lacking Leu, Trp, His and adenine (Ade) in the presence of 5-bromo-4-chloro-3-indolyl-α-d-galactopyranoside (X-α-Gal) (Sigma).

### Glutaraldehyde cross-linking of proteins

For the protein oligomerization assays, Sf9 cells were transfected with pDHsp/VP19-V5-His, pDHsp/VP24-V5-His or pDHsp/VP51A-V5-His plasmid DNA and heat shocked as described above. The transfected cells were then washed with PBS, lysed in a hypotonic buffer (10 mM Tris-HCl [pH 7.5], 10 mM KCl, and 5 mM MgCl_2_), and incubated on ice for 20 min. The swollen cells were passed through a 25-gauge needle 20 times to disrupt the cells. After centrifugation at 1,000×*g*, the supernatant was incubated with glutaraldehyde (Sigma) at a final concentration of 0.01% at room temperature for various time periods. The reactions were stopped by the addition of an equal volume of 2× SDS sample buffer, and the samples were subjected to Western blotting using anti-V5 antibody.

### Identification of VP19, VP24, VP26, VP28 and VP51A interaction domains

In order to elucidate the interaction domains for each pair of interactions, truncated VP19, VP24, VP26, VP28 and VP51A proteins were subjected to coimmunoprecipitation assays. Since these truncated proteins would be too small to be analyzed directly, partial fragments of the VP19, VP24, VP26, VP28 and VP51A genes were cloned into a pDHsp/EGFP-V5-His expression vector. This vector was modified from pDHsp/V5-His by inserting an EGFP (enhanced green fluorescence protein) gene (derived from the pEGFP-N1 vector, Clontech) in a multiple cloning site downstream of the *Drosophila* heat shock protein 70 gene promoter. The resulting constructs were then used to express the corresponding chimeric proteins, with EGFP serving to increase the molecular mass of the truncated proteins. The VP19, VP24, VP26 and VP28 constructs were designed to express C- or N-terminal truncated mutants, while the larger VP51A gene was cloned in three separate parts. Accordingly, the following plasmids were constructed: pDHsp/EGFP-VP19_1–66_-V5-His, pDHsp/EGFP-VP19_62–121_-V5-His, pDHsp/EGFP-VP24_1–105_-V5-His, pDHsp/EGFP-VP24_99–208_-V5-His, pDHsp/EGFP-VP26_1–97_-V5-His, pDHsp/EGFP-VP26_85–204_-V5-His, pDHsp/EGFP-VP28_1–133_-V5-His, pDHsp/EGFP-VP28_110–204_-V5-His, pDHsp/EGFP-VP51A_1–165_-V5-His, pDHsp/EGFP-VP51A_168–333_-V5-His, pDHsp/EGFP-VP51A_326–448_-V5-His. The suffix numbers of each plasmid indicate the amino acid positions spanned by the cloned gene fragments. The primers used to construct these plasmids are listed in Table S1. The plasmids containing these partial fragments were then used in coimmunoprecipitation assays in which plasmids containing a full length FLAG-tagged structural protein gene were cotransfected into the same Sf9 insect cells. Only combinations of proteins that were known to interact were used in these assays. Known interactions between WSSV structural proteins are summarized in Table 1.

### Electron microscopy of the WSSV virion

Following the method of Tsai et al. [7], aliquots (10 µl) of suspension of purified intact WSSV virions and nucleocapsids were adsorbed onto Formvar-supported grids (200 mesh) for 5 min at room temperature and then the excess solution was removed. The grids were then stained with 2% phosphotungstic acid (pH 7.4) for 30 sec. Specimens were examined with a transmission electron microscope JEOL JEM1010.

### Membrane topology prediction and 3D models

The amino acid sequence for VP19 (GI:19482065), VP24 (GI:19481650), VP26 (GI:19481959), VP28 (GI:19482072) and VP51A (GI:19481886) were downloaded from GenBank (http://ncbi.nlm.nih.gov). The web-versions of three different topology prediction methods were used to model the topology of these five envelope proteins: SOSUI (http://bp.nuap.nagoya-u.ac.jp/sosui/sosuiframe0.html) [52], TMHMM (http://www.cbs.dtu.dk/services/TMHMM-2.0/) [53], and TMpred (http://bioweb.pasteur.fr/seqanal/interfaces/toppred.html) [54]. All these methods were used in single sequence mode and all user adjustable parameters were left at their default values. Graphics of the 3D model of the viral protein complex were produced with UCSF Chimera [55] using the solved structures for WSSV VP26 and VP28 (PDB:2edm and 2ed6, respectively [39]) and the trans-membrane helix structure (PDB:1h2s) as references. A 3D model of the nucleocapsid was also constructed based on electron microscopy of the negatively stained WSSV virion (Figure 11B) and the molecular mass of the major nucleocapsid protein VP664.

## Supporting Information

Table S1 Primer sequences used for the construction of various expression plasmids.
(0.06 MB DOC)
