# Supplementary material for: A 3D Model of the Membrane Protein Complex Formed by the White Spot Syndrome Virus Structural Proteins
Source: PLoS One. 2010 May 19;5(5):e10718. doi: 10.1371/journal.pone.0010718 (PMC2873410; doi:10.1371/journal.pone.0010718)
Supplement: Table S1 — Primer sequences used for the construction of various expression plasmids. (0.06 MB DOC) [file pone.0010718.s001.doc]

Table S1. Primer sequences used for the construction of various expression plasmids

| Construct | Primer*^a^* | Sequences (5′-3′)*^b^* |
| --- | --- | --- |
| pDHsp/VP19-FLAG-His  pDHsp/VP19-V5-His | F  R | CCCAAGCTTAAAAAAATGGCCACCACGACTAAC  TCCCCGCGGCTGCCTCCTCTTGGGGTAAGAC |
| pDHsp/VP24-FLAG-His  pDHsp/VP24-V5-His | F  R | CCCAAGCTTTTCAAAATGCACATGTGGGGG  TCCCCGCGGTTTTTCCCCAACCTTAAACAG |
| pDHsp/VP26-FLAG-His  pDHsp/VP26-V5-His | F  R | CCCAAGCTTAGAAAAATGGAATTTGGCAAC  TCCCCGCGGCTTCTTCTTGATTTCGTCCTTG |
| pDHsp/VP28-FLAG-His  pDHsp/VP28-V5-His | F  R | CCCAAGCTTCTCGTCATGGATCTTTCTTTC  TCCCCGCGGCTCGGTCTCAGTGCCAGAGTAG |
| pDHsp/VP51A-FLAG-His  pDHsp/VP51A-V5-His | F  R | CCCAAGCTTGAAAAAATGTTCGTCATAAGC  TCCCCGCGGTTGGCTGGACAATAAATTTTTG |
| pGAD-VP19 | F  R | CGGAATTCATGGCCACCACGACTAACAC  CCGCTCGAGGCTGCCTCCTCTTGGGGTAAG |
| pGAD-VP24 | F  R | CGGAATTCATGCACATGTGGGGGGTTTAC  CCGCTCGAGGTTTTTCCCCAACCTTAAACA |
| pGAD-VP26 | F  R | CGGAATTCATGGAATTTGGCAACCTAAC  CCGCTCGAGGCTTCTTCTTGATTTCGTCCT |
| pGAD-VP51A | F  R | TCCCCCGGGAATGTTCGTCATAAGCATAGC  CGGGATCCTTATTGGCTGGACAATAAATT |
| pDHsp/EGFP-V5-His | F  R | CCCAAGCTTACCATGGTGAGCAAGGGCGAGGAG  CGCGGATCCCTTGTACAGCTCGTCCATGCC |
| pDHsp/EGFP-VP19_1-66_-V5-His | F  R | CGCGGATCCATGGCCACCACGACTAACAC  TCCCCGCGGAGAATCGCTGTCCTTCTTTG |
| pDHsp/EGFP-VP19_62-121_-V5-His | F  R | CGCGGATCCGACAGCGATTCTGACAC  TCCCCGCGGCTGCCTCCTCTTGGGGTAAG |
| pDHsp/EGFP-VP24_1-105_-V5-His | F  R | CGCGGATCCATGCACATGTGGGGGGTT  TCCCCGCGGTCCGTCTCCAAGTAAAGATG |
| pDHsp/EGFP-VP24_99-208_-V5-His | F  R | CGCGGATCCTCTTTACTTGGAGACGG  TCCCCGCGGTTTTTCCCCAACCTTAAACA |
| pDHsp/EGFP-VP26_1-97_-V5-His | F  R | CGCGGATCCATGGAATTTGGCAACCTAAC  TCCCCGCGGAGAGATGACAAGATCAGCAG |
| pDHsp/EGFP-VP26_85-204_-V5-His | F  R | CGCGGATCCGACATGAAGGATGTTTC  TCCCCGCGGCTTCTTCTTGATTTCGTCCTT |
| pDHsp/EGFP-VP28_1-133_-V5-His | F  R | CGCGGATCCATGGATCTTTCTTTCACTC  TCCCCGCGGGTTGATCTTTCTTGATGTGTTG |
| pDHsp/EGFP-VP28_110-204_-V5-His | F  R | CGCGGATCCACTGTGGGGCAGAATCTC  TCCCCGCGGCTCGGTCTCAGTGCCAGAG |
| pDHsp/EGFP-VP51A_1-165_-V5-His | F  R | CGCGGATCCATGTTC GTCATAAGCATAGC  TCCCCGCGGTTTAGGTTTTGAAGATATGG |
| pDHsp/EGFP-VP51A_168-333_-V5-His | F  R | CGCGGATCCGAATACTATGAAGCATTCGT  TCCCCGCGGGTACTGCATAGTGTTTGAAG |
| pDHsp/EGFP-VP51A_326-448_-V5-His | F  R | CGCGGATCCTCAAACACTATGCAGTACAG  TCCCCGCGGTTGGCTGGACAATAAATTTT |

*^a^* F, forward; R, reverse.

*^b^* The restriction enzyme cutting sites are underlined.
